# A New Bromoallene-Producing Chemical Type of the Red Alga *Laurencia nangii* Masuda

**DOI:** 10.3390/molecules17022119

**Published:** 2012-02-21

**Authors:** Takashi Kamada, Charles Santhanaraju Vairappan

**Affiliations:** Laboratory of Natural Products Chemistry, Institute for Tropical Biology and Conservation, Universiti Malaysia Sabah, 88400 Kota Kinabalu, Sabah, Malaysia; Email: takashi.k.sugisawa@gmail.com

**Keywords:** *Laurencia nangii*, C_15_-acetogenin, bromoallene, chemical race

## Abstract

Six populations of *Laurencia nangii* were found to produce three bromoallenes; dihydroitomanallene B (**1**), itomanallene B (**2**) and pannosallene (**3**). Prior to this report, *L. nangii* were only known to produce C_15_-acetogenins with acetylene functionality. This could be regarded as a new chemical race of *L. nangii*. The compound structures were elucidated on the basis of spectroscopic analysis and comparison with those previously reported in literature. Compound **1**, dihydroitomanallene B, was isolated as a new compound representing a minor variation of itomanallene B (**2**).

## 1. Introduction

The Red algal genus *Laurencia* (Rhodomelaceae, Ceramiales) is a prolific producer of halogenated secondary metabolites such as sesquiterpenes, diterpenes, triterpenes and C_15_-acetogenins [1]. Species of this genus are known to produce characteristic sets of halogenated secondary metabolites [2]. However, some species have been reported to produce related or unrelated sets of halogenated metabolites in different populations that are geographically close or distant [3,4]. Several morphologically similar, but chemically distinct, populations have been found in *Laurencia nipponica* Yamada and *Laurencia majuscula* (Harvey) Lucas growing in Japan. Thus, the existence of different chemical types of the same species had been suggested as chemical races [2].

In an ongoing investigation pertaining to the chemical constituents of red algae genus *Laurencia* from the coastal waters of Borneo (Malaysia), we reported the chemical composition of *L. snackeyi* (Weber-van Bosse) Masuda [5], *L. similis* Nam *et* Saito [6], *L. nangii* (Masuda) [7], *L. majuscula* (Harvey) Lucas [8,9,10,11] and *Laurencia* species [12,13]. Recently, we collected and examined six populations of *L. nangii* from Tun Sakaran Marine Park, Sabah, Malaysia. These six populations contained different types of halogenated non-terpene metabolites that led us to suggest the presence of “chemical races” in *L. nangii* of North Borneo Island, Sabah. Each of these populations showed the presence of a new bromoallene [dihydroitomanallene B (**1**)] along with two known bromoallenes, itomanallene B (**2**) and pannosallene (**3**). The structure of the new compound, dihydroitomanallene B (**1**), was very similar to the known itomanallene B (**2**) and was elucidated based on spectral data. The structures of known metabolites **2**–**3** were determined based on the comparison of spectral data of published reports of Suzuki *et al.* [14] and Suzuki *et al.* [15]. In this paper, we report the discovery of a bromoallene-producing *L. nangii* and the structure of compound **1**. Compounds **1** and **2** were very labile in CDCl_3_, therefore their spectroscopic data were taken in C_6_D_6_. Hence, this paper will describe the isolation and structure elucidation of compound **1** and the importance of bromoallenes as chemotaxonomical markers in the red alga *L. nangii*.

## 2. Results and Discussion

Compound **1** was isolated as a colorless oil, [α]^25^_D_ + 64.01° (CHCl_3_). HR-MS gave a molecular formula of C_17_H_25_BrO_3_. The ^1^H- and ^13^C-NMR signals of **1** showed the presence of a typical terminal bromoallene moiety at δH 5.66 (1H, dd, *J* = 5.8, 2.0 Hz) and 5.32 (1H, dd, *J* = 5.8, 5.8 Hz); δ_C_ 201.5 (C), 102.6 (CH) and 73.5 (CH). The IR spectrum revealed the presence of an acetoxyl group without any hydroxyl groups at *v*_max_ 1,720 cm^−1^, which was supported by a methyl signal at δH 1.67 (3H, s) in the ^1^H-NMR spectrum. Detailed ^1^H and ^13^C-NMR data are given in Table 1. It is also important to note that data presented in Table 1 was taken in C_6_D_6_ because **1** was easily decomposed when spectra were taken in CDCl_3_. However, data comparison of **2** with that of Suzuki *et al.* [14] was done in CDCl_3_ since **2** was stable in this solvent. Chemical shift data of **1** and **2** taken in C_6_D_6_ are shown in Table 1.

**Figure 1:**
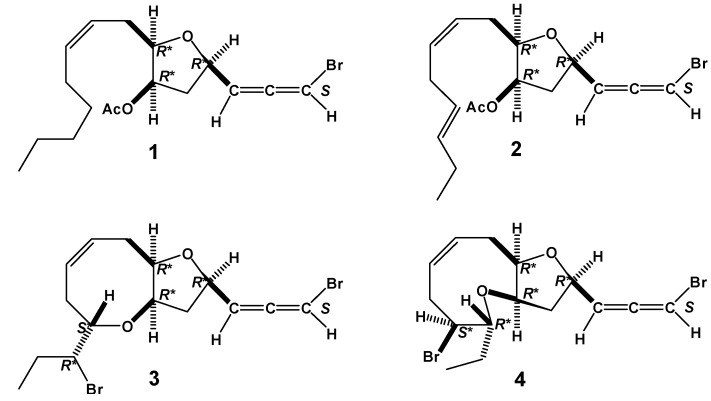
Bromoallene compounds **1**, **2**, **3** and **4**.

The planar structure of **1** was readily determined as formula **1** (Figure 1) by detailed analysis of ^1^H- and ^13^C-NMR, ^1^H-^1^H COSY, HSQC and HMBC spectral data. Moreover, the close resemblance between the C1–C10 ^1^H and ^13^C-NMR data of **1** and **2**, together with co-existence of **1** and **2** in same alga indicated that **1** has the same chiral centers at C4, C6 and C7 as **2** and also the double bond at C9–C10 is in the *Z*-configuration. The bromoallenic moiety of **1** was also assigned as *S* from the strong positive rotation by application of Lowe’s rule [16].

**Scheme 1:**
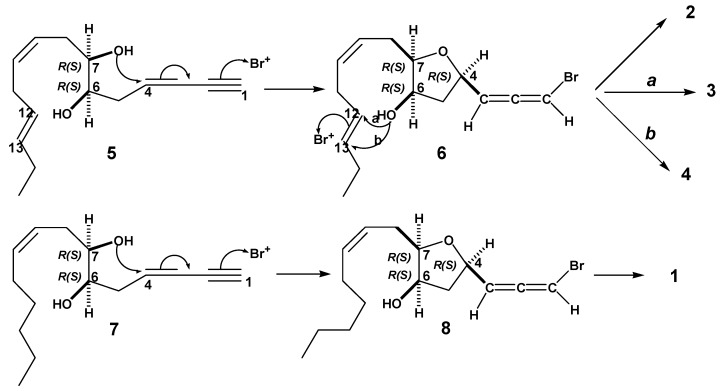
Biogenesis pathway of compounds **1**, **2**, **3** and **4**.

The co-existence of dihydroitomanallene B (**1**), itomanallene B (**2**) and pannosallene (**3**) in Malaysian *L. nangii*, and itomanallene B (**2**) and itomanallene A (**4**) in Japanese *Laurencia intricate* Lamouroux strongly suggested that these four bromoallenes would have the same absolute configurations at C4, C6 and C7. The halogenated C_15_-acetogenins isolated from various *Laurencia* have been assumed to arise from common precursors, (6*R*,7*R*) or (6*S*,7*S*)-laurediol (**5**) [17]. Hence, dihydroitomanallene B (**1**) may arise from (6*R*,7*R*) or (6*S*,7*S*)-12,13-dihydrolaurediol (**6**) [18]. Bromonium ion-catalyzed cyclization between the hydroxyl group at C7 and C4 in (6*R*,7*R*) or (6*S*,7S)-laurediol (**5**) would give a monocyclic bromoallene (**6**) with (4*R*,6*R*,7*R*) or (4*S*,6*S*,7*S*)-configuration. As described by Kikuchi *et al.* [19], the configurations between C12 and C13 of C_15_-acetogenins are (12*R*,13*S*) or (12*S*,3*R*)-erythro, reflecting the (12*E*)-double bond in both precursors, (6*R*,7*R*)-laurediols and (6*S*,7*S*)-laurediols. Pannosallene (**3**) and itomanallene A (**4**) could be biosynthesized from **6** by (12*R*,13*R*)-bromonium ion-catalyzed cyclization, *via* route ***a*** and ***b*** respectively, as shown in Scheme 1. Compound **6** would further afford itomanallene B (**2)** via acetylation. Similarly, bromonium ion-catalyzed cyclization between the hydroxyl group C7 and C4 in (6*R*,7*R*) or (6*S*,7*S*)-12,13-dihydrolaurediol (**7**) would give a bromoallene (**8**), which would lead to the formation of dihydroitomanallene B (**1**) via acetylation. Thus, the absolute configurations of dihydroitomanallene B (**1**) and itomanallene B (**2**) would be 4*R*, 6*R* and 7*R* or 4*S*, 6*S* and 7*S* as in the case of pannosallene (**3**) and itomanallene A (**4**). The relative configuration between C3 and C4 remains unclear.

There are close to 50 species of *Laurencia* in this genus, that could be distinguished based on their morphological features and the type of halogenated metabolites they produce. Approximately 500 types of halogenated metabolites have been isolated and they are useful chemotaxonomic markers in taxonomical classification of this genus [1,16,19]. In the Malaysian coastal waters, there are four major species of *Laurencia*; *L. snackeyi*, *L. majuscula*, *L. similis* and *L. nangii*. They are morphologically distinguishable and could be identified by the halogenated secondary metabolites they produce. *Laurencia snackeyi* produces halogenated snyderane sesquiterpenes, *L. majuscula* produces halogenated chamigranes, while, *L. similis* produces polybrominated indoles [5,6,8,9,10]. 

**Figure 2:**
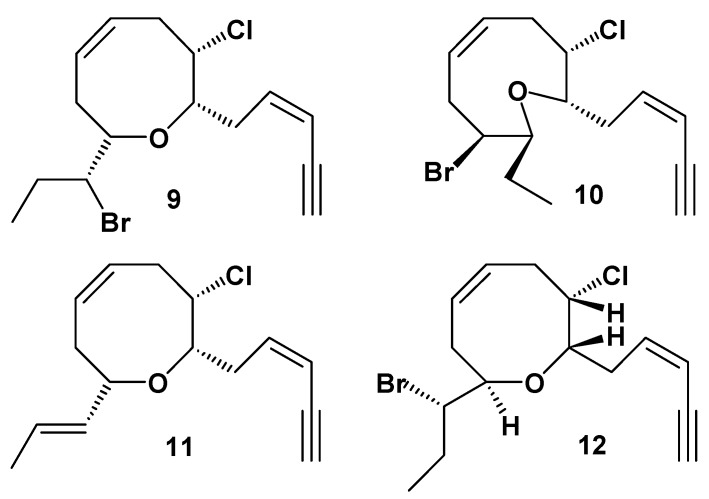
C_15_-acetogenin compounds **9**, **10**, **11** and **12**.

Information on the chemistry of *L. nangii* is very scarce and only two other publications are available, both reporting C_15_-acetogenins with acetylenic functionalities as the halogenated metabolites produced [7,20]. Masuda originally described a type of *L. nangii* based on several specimens collected from Vietnam. It is a tropical alga with a wide distribution in the South East Asian waters, has been reported to be growing wild in Vietnam, Philippines, Indonesia and Malaysia [21]. During our routine field collection, we discovered six populations of *L. nangii* in Tun Sakaran Marine Park (South East of North Borneo Island) that produces these three bromoallenes. Compound **1**, dihydroitomanallene B, is a new unstable compound with minor chemical differences with itomanallene B (**2**). Itomanallene B (**2**) was first isolated from *L. intricate* by Suzuki *et al*. [14], while panasallene (**3**) was isolated from *Laurencia pannosa* Zanardini collected from Vietnam [15]. Both these specimen are morphologically different from *L. nangii*. Two other reports on *L. nangii*, reported C_15_-acetogenins, *cis*-pinnatifidenyne (**9**), obtusenyne (**10**), 3(*Z*)-laurenyne (**11**) and cis-dihydrorhodophytin (**12**) (Figure 2) as its halogenated metabolite [7,19]. Masuda *et al*. also reported aplysiadiol as a constituent of *L. nangii*. However, upon reexamination, it was apparent that it was a contamination from *Laurencia* sp. that was found growing between thallus of *L. nangii*. To the best of our knowledge, this is the first report of a type of *L. nangii* that only produces bromoallene as its secondary metabolites. Hence, it is suggested this could be a new chemical race of *L. nangii*.

## 3. Experimental

### 3.1. General

Optical rotations were measured on an AUTOPOL IV automatic polarimeter (Rudolph Research Analytical, Hackettstown, NJ, USA). ^1^H-NMR (600 MHz) and ^13^C-NMR (150 MHz) spectra were recorded with a JEOL ECA 600, with TMS as internal standard. HR-ESI-TOFMS spectrum was obtained with LCMS-IT-TOF (Shimadzu, Kyoto, Japan). Preparative TLC was performed with silica gel plate (Merck, Frankfurt, Germany; Kieselgel 60 F_254_). Silica gel (Merck, Kieselgel 60, 70–230 mesh) was used for column chromatography. Analytical TLC was performed on Merck Kieselgel 60 F_254_. Spots were visualized by UV light or by spraying with a 5% phosphomolybdic acid-ethanol solution.

### 3.2. Biological Material

The specimen of *L. nangii* was collected from Tun Sakaran Marine Park, Sabah (5°19'58"N, 115°12'02"E), between 12–28 October 2010. Collected specimens were transported at 4 °C, and air-dried at the laboratory. The voucher herbariums (MAR45943BOR) were made and deposited in the BORNEENSIS Collection of Institute for Tropical Biology and Conservation, Universiti Malaysia Sabah.

### 3.3. Extraction and Isolation

The air-dried *Laurencia* (400 g) was extracted with MeOH (1 L) at room temperature for 7 days. The crude extract was evaporated under reduced pressure and the residue was partitioned between EtO_2_ and H_2_O. The EtO_2_ fraction was further exposed to anhydrous sodium sulphate to remove moisture, and concentrated *in vacuo* to yield a dark green crude extract. The EtO_2_ extract (800 mg) was chromatographed on a Si gel column using a hexane and EtOAc gradient of increasing polarity (hex:EtOAc; 9.5:0.5, 9:1, 8:2, 7:3, 6:4, 1:1) as eluant to yield six fractions. A portion of fraction 2 (85.8 mg) eluted with hexane/EtOAc (8:2) was submitted to repeated preparative TLC with CHCl_3_ and toluene to yield compounds **1** (8.4 mg), **2** (5.2 mg) and **3** (6.8 mg).

### 3.4. Dihydroitomanallene B (**1**)

Colorless oil; [α]^25^_D_ + 64.01° (CHCl_3_; *c* 0.39); IR *v*_max_ (CHCl_3_) cm^−1^: 2910, 1720, 1350, 1240, 1010, 800; LR-EIMS *m/z* (rel. int): 315, 313 (3:3) [M–CH_3_CO]^+^, 275 (32), 273 (32), 247, 245 (16:16) [M-C_8_H_15_]^+^, 217 (18), 111 (25), 43 (100). HR-TOFMS *m*/*z* 356.0194 [M]^+^ (calcd. for C_17_H_25_^79^BrO_3_, 356.0187); ^1^H-NMR and ^13^C-NMR spectral data: see Table 1.

**Table 1:** ^1^H-NMR and ^13^C-NMR spectral data of compound **1** (recorded at 600/150 MHz in C_6_D_6_; δ in ppm, *J* in Hz).

C	1		2	
	^13^C	^1^H ( *J* in Hz)	^13^C	^1^H ( *J* in Hz)
1	73.5	5.66 (dd, *J* = 5.8, 2.0 Hz, 1H)	73.6	5.67 (dd, *J* = 5.8, 2.0 Hz, 1H)
2	201.5	-	202.5	-
3	102.6	5.32 (dd, *J* = 5.8, 5.8 Hz, 1H)	102.6	5.31 (dd, *J* = 5.8, 5.8 Hz, 1H)
4	74.0	4.16 (dddd, *J* = 7.3, 5.8, 5.4, 2.0, 1H)	74.0	4.15 (dddd, *J* = 7.3, 5.8, 5.4, 2.0, 1H)
5	38.9	1.92 (ddd, *J* = 13.7, 5.4, 1.5 Hz, 1H)	38.9	1.90 (ddd, *J* = 13.7, 5.4, 1.5 Hz, 1H)
		1.67 (m, 1H)		1.65 (m, 1H)
6	73.9	5.08 (m, 1H)	73.9	5.04 (m, 1H)
7	81.8	3.47 (ddd, *J* = 7.3, 7.3, 3.4 Hz, 1H)	81.6	3.44 (ddd, *J* = 7.3, 7.3, 3.4 Hz, 1H)
8	27.5	2.54 (ddd, *J* = 13.7, 7.3, 3.4 Hz, 1H)	27.3	2.51 (ddd, *J* = 13.7, 7.3, 3.4 Hz, 1H)
		2.46 (ddd, *J* = 13.7, 7.3, 3.4 Hz, 1H)		2.43 (ddd, *J* = 13.7, 7.3, 3.4 Hz, 1H)
9	124.9	5.49 (m, 1H)	125.5	5.50 (m, 1H)
10	132.1	5.49 (m, 1H)	130.0	5.54 (m, 1H)
11	27.4	2.02 (m, 2H)	30.5	2.75 (br t, *J* = 6.8 Hz, 2H)
12	29.4	1.30 (m, 2H)	127.0	5.39 (m, 1H)
13	31.5	1.22 (m, 2H)	132.4	5.45 (m, 1H)
14	22.7	1.24 (m, 2H)	25.6	1.94 (m, 2H)
15	13.9	0.87 (t, *J* = 7.3 Hz, 3H)	13.7	0.92 (t, *J* = 7.3 Hz, 3H)
OAc	169.5	-	170.3	-
	20.4	1.67 (s, 1H)	20.4	1.66 (s, 1H)

## 4. Conclusions

As a part of our chemical investigation on the Bornean red algae genus *Laurencia*, a new chemical race of *L. nangii* is reported for the first time. A new compound with minor variation from itomanallene B (**2**) was isolated and identified as dihydroitomanallene B (**1**). A total of three bromoallenes [dihydroitomanallene B (**1**), itomanallene B (**2**) and pannosallene (**3**)] were isolated and identified from six populations of *L. nangii* collected from Tun Sakaran Marine Park, Semporna, Sabah, Malaysia. This finding has enriched our knowledge on the chemical constituents of Borneon red algae genus *Laurencia*. Since, chemical race populations of *L. nangii* have not been reported to date, this is the first such finding and suggests this species to consist of at least two chemical races; one that produces C_15_ acetylenes and another that produces C_15_ bromoallenes.

## Supplementary Materials

Supplementary materials can be accessed at: http://www.mdpi.com/1420-3049/17/2/2119/s1 .
