# Supplementary material for: A New Bromoallene-Producing Chemical Type of the Red Alga Laurencia nangii Masuda
Source: Molecules. 2012 Feb 21;17(2):2119–25. doi: 10.3390/molecules17022119 (PMC6290570; doi:10.3390/molecules17022119)
Supplement: Supplementary file 1 [file molecules-17-02119-s001.pdf]

SUPPLEMENT DATA

COMPOUND-1

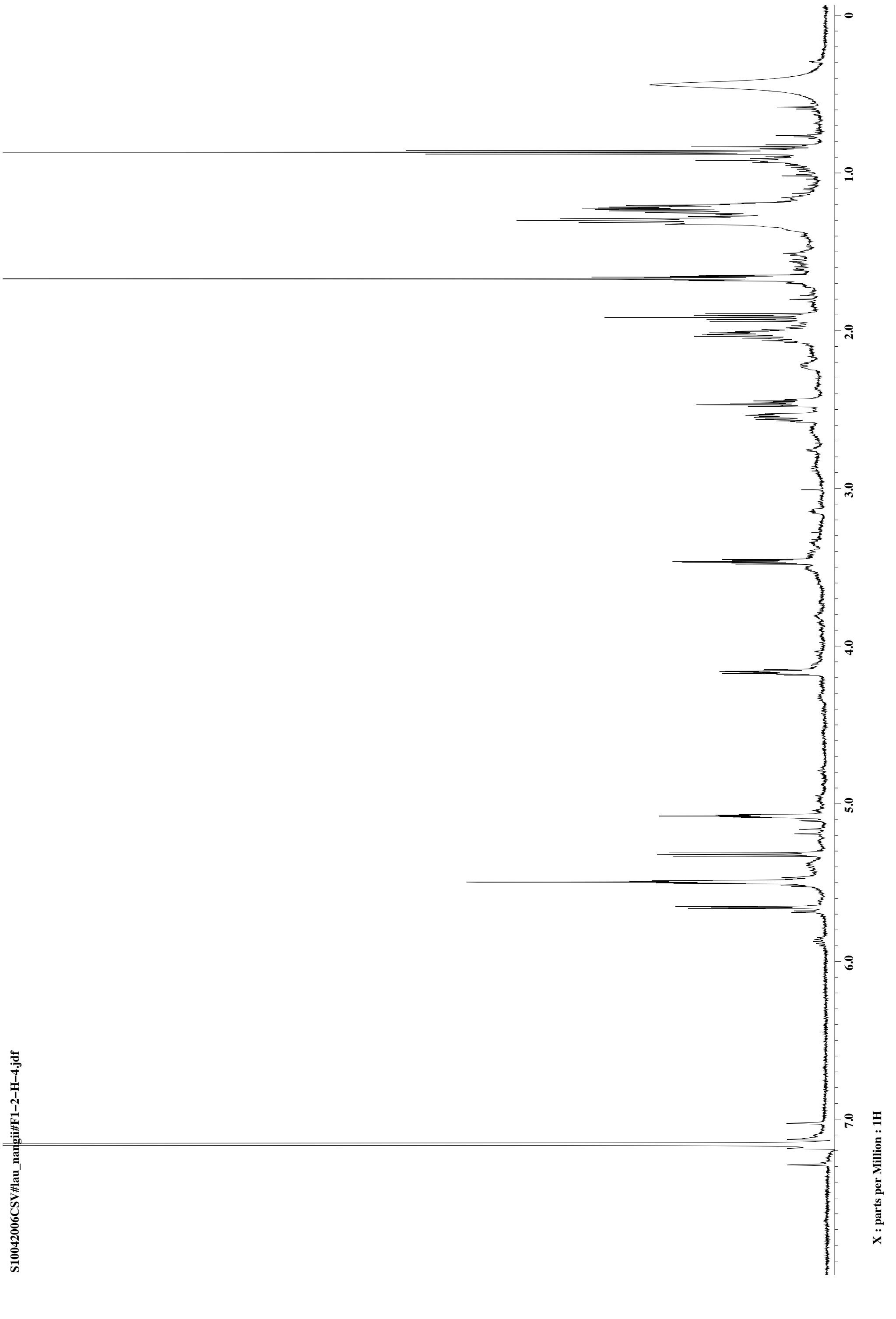

H-NMR COMPOUND 1

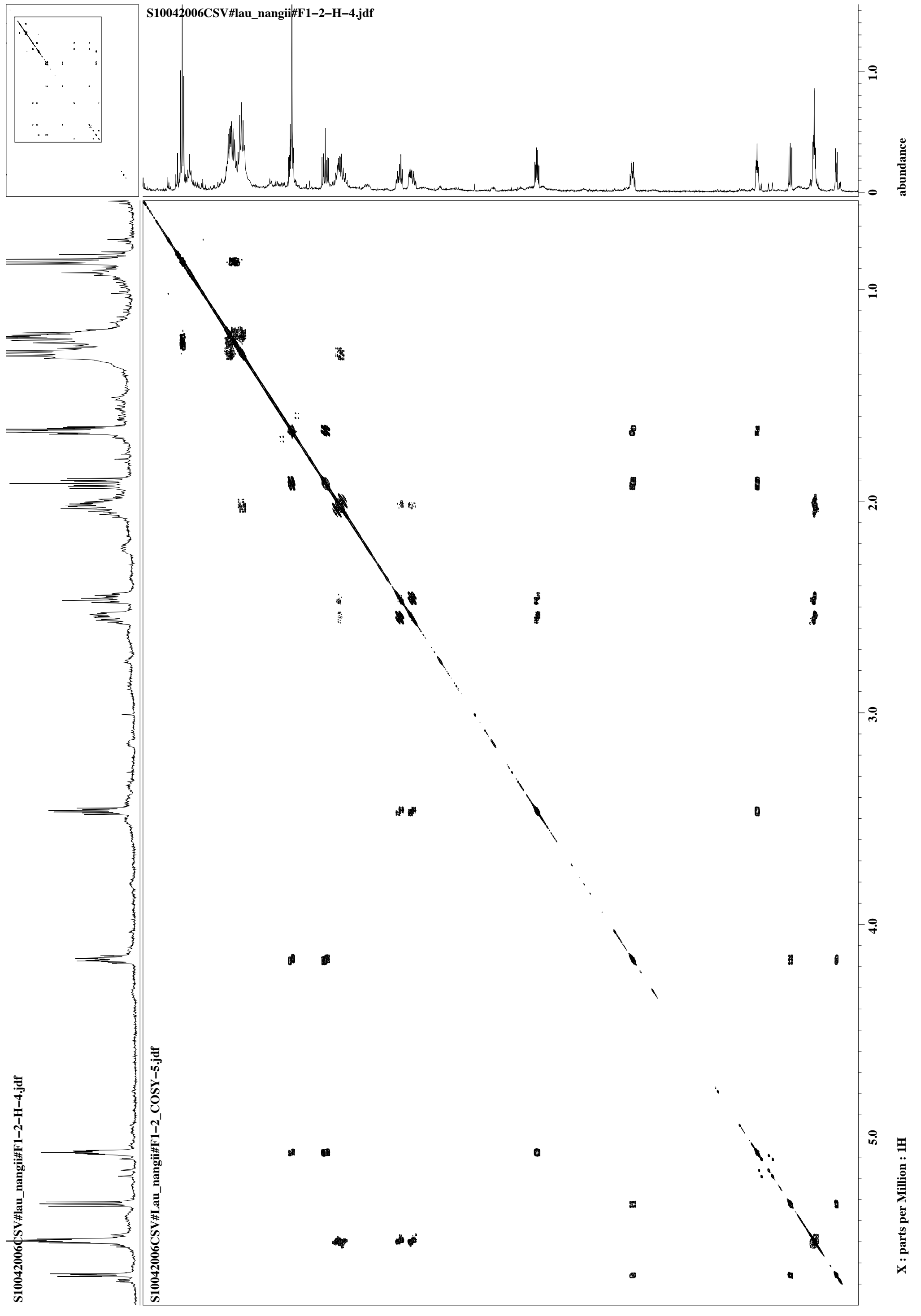

H-H- COSY COMPOUND 1

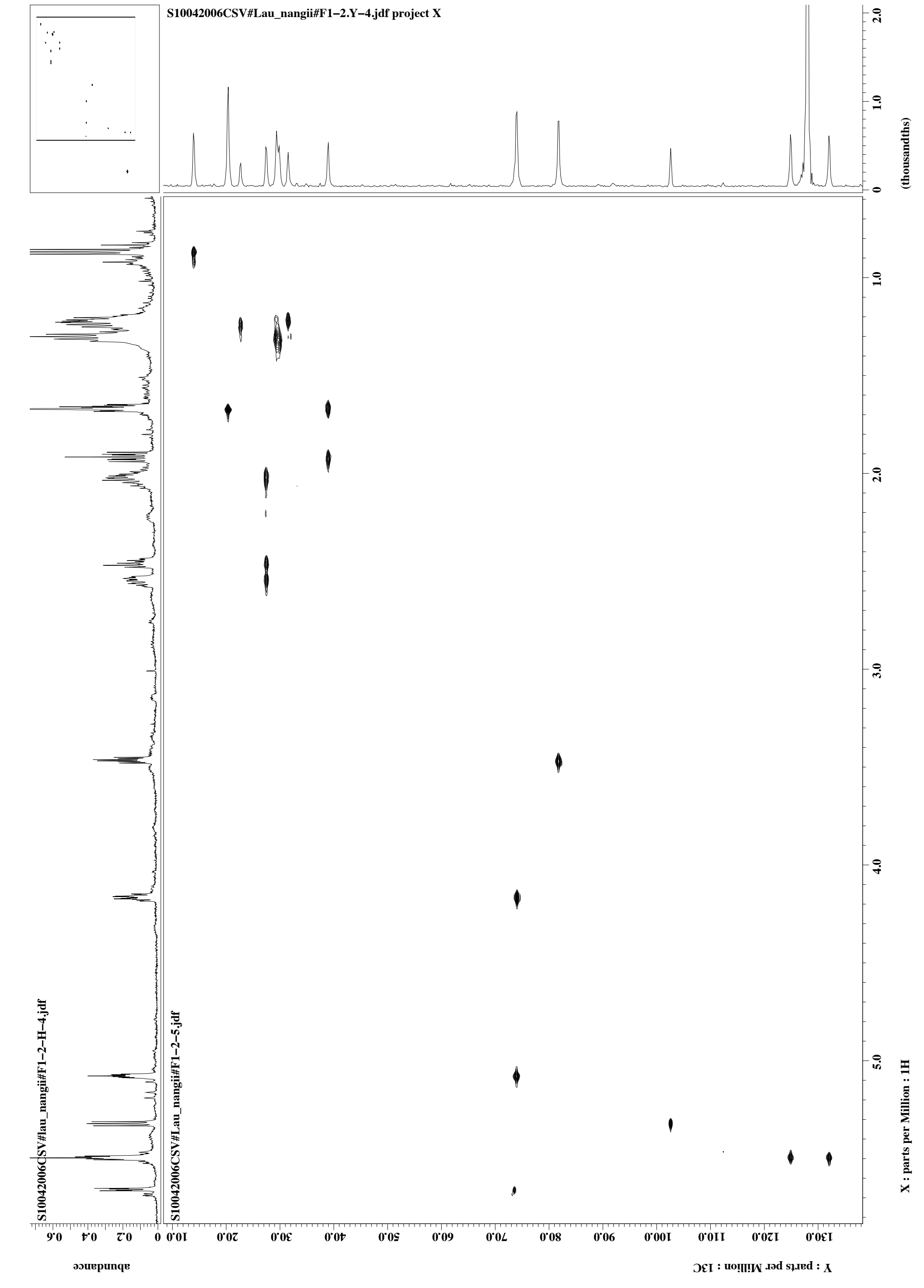

HSQC COMPOUND 1

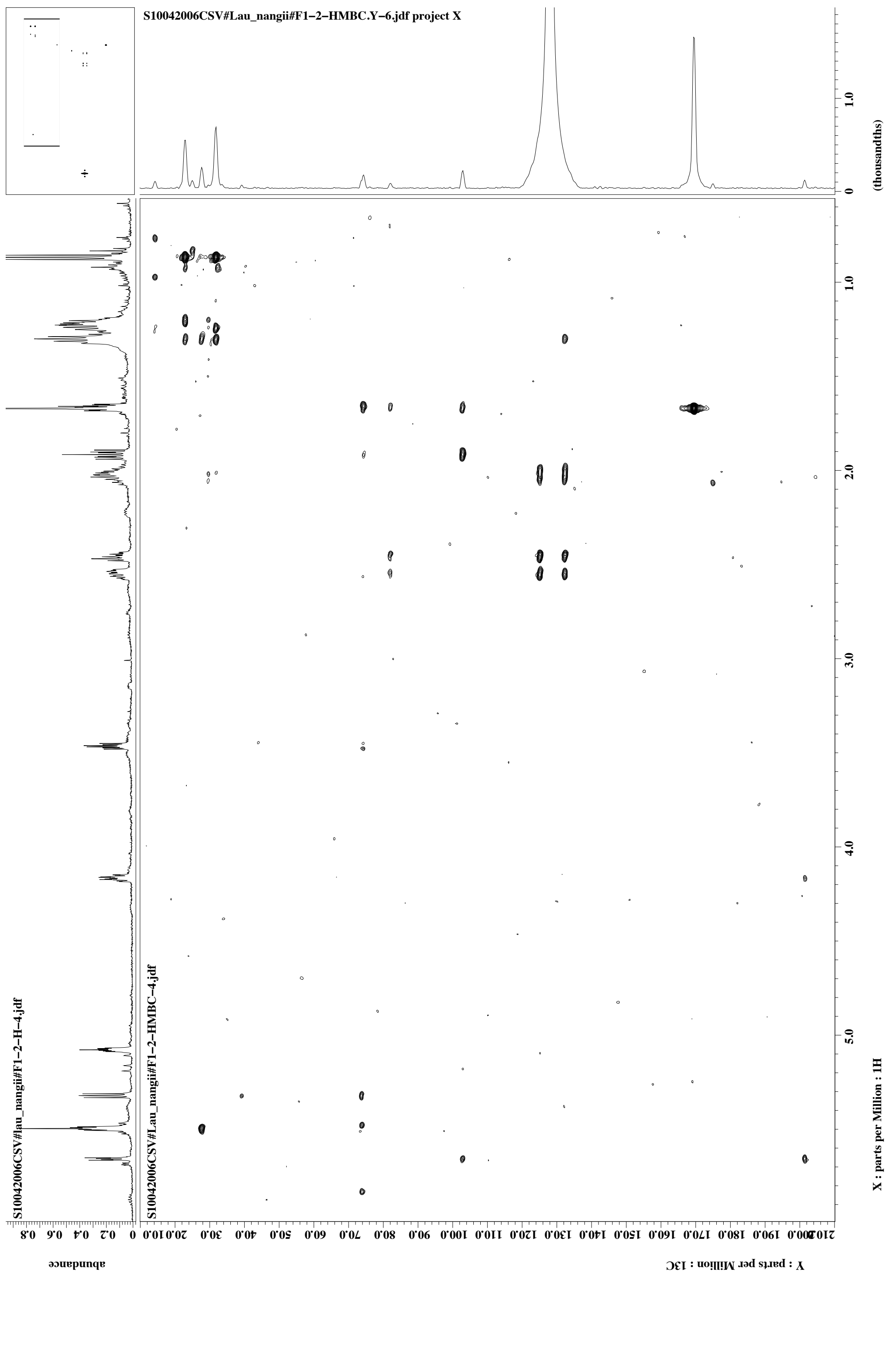

HMBC COMPOUND 1

COMPOUND-2

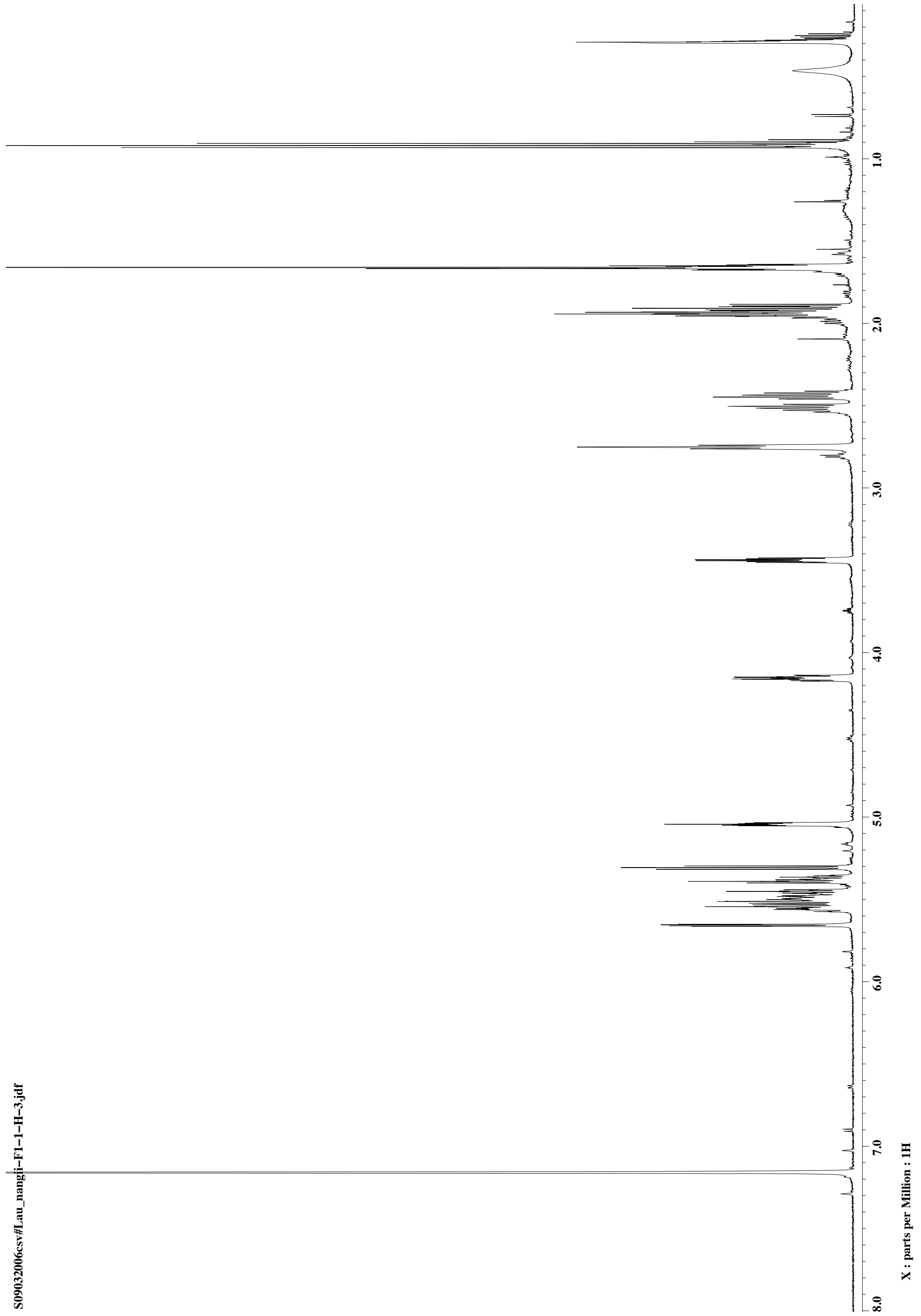

H-NMR COMPOUND 2

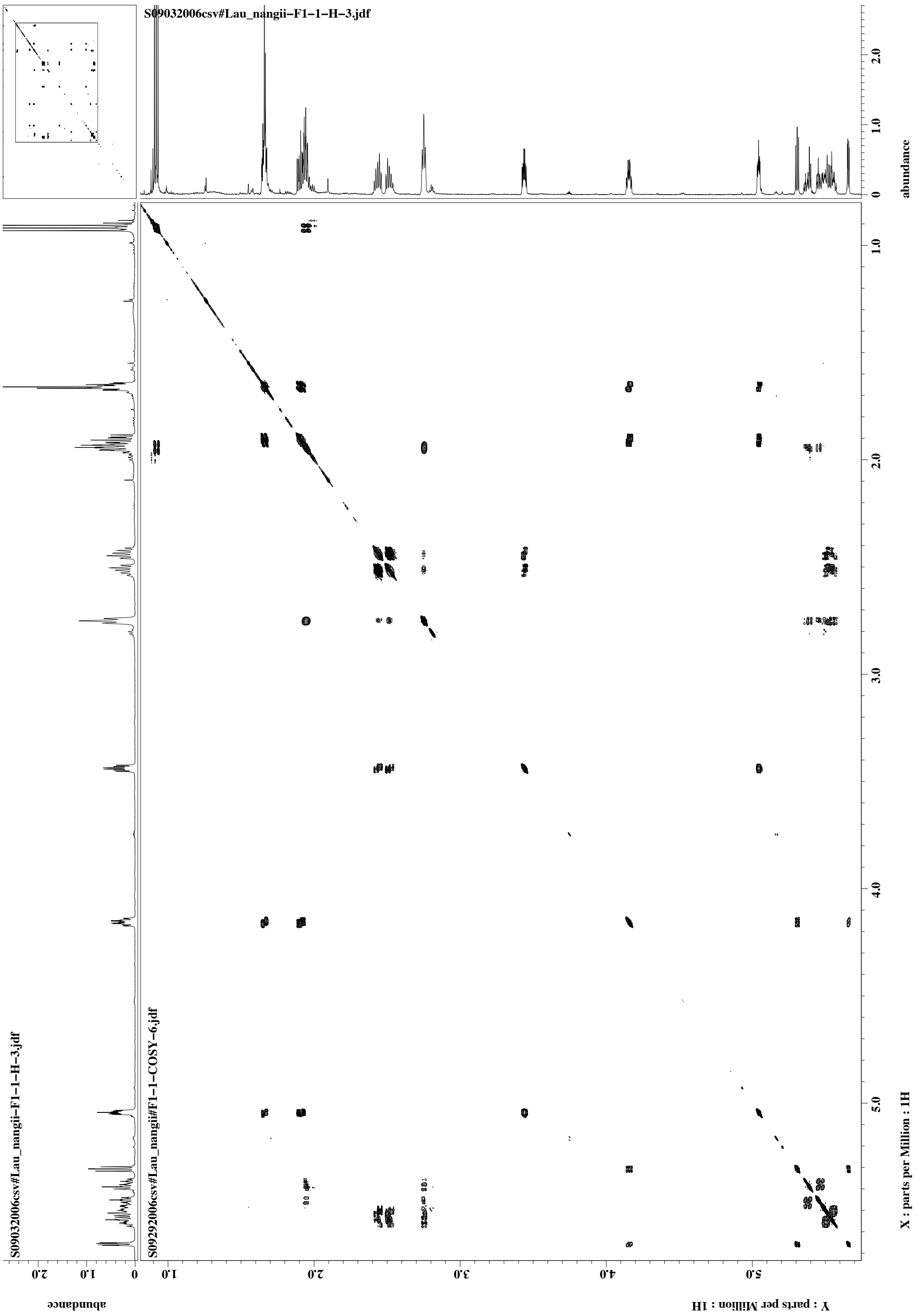

H-H-COSY COMPOUND 2

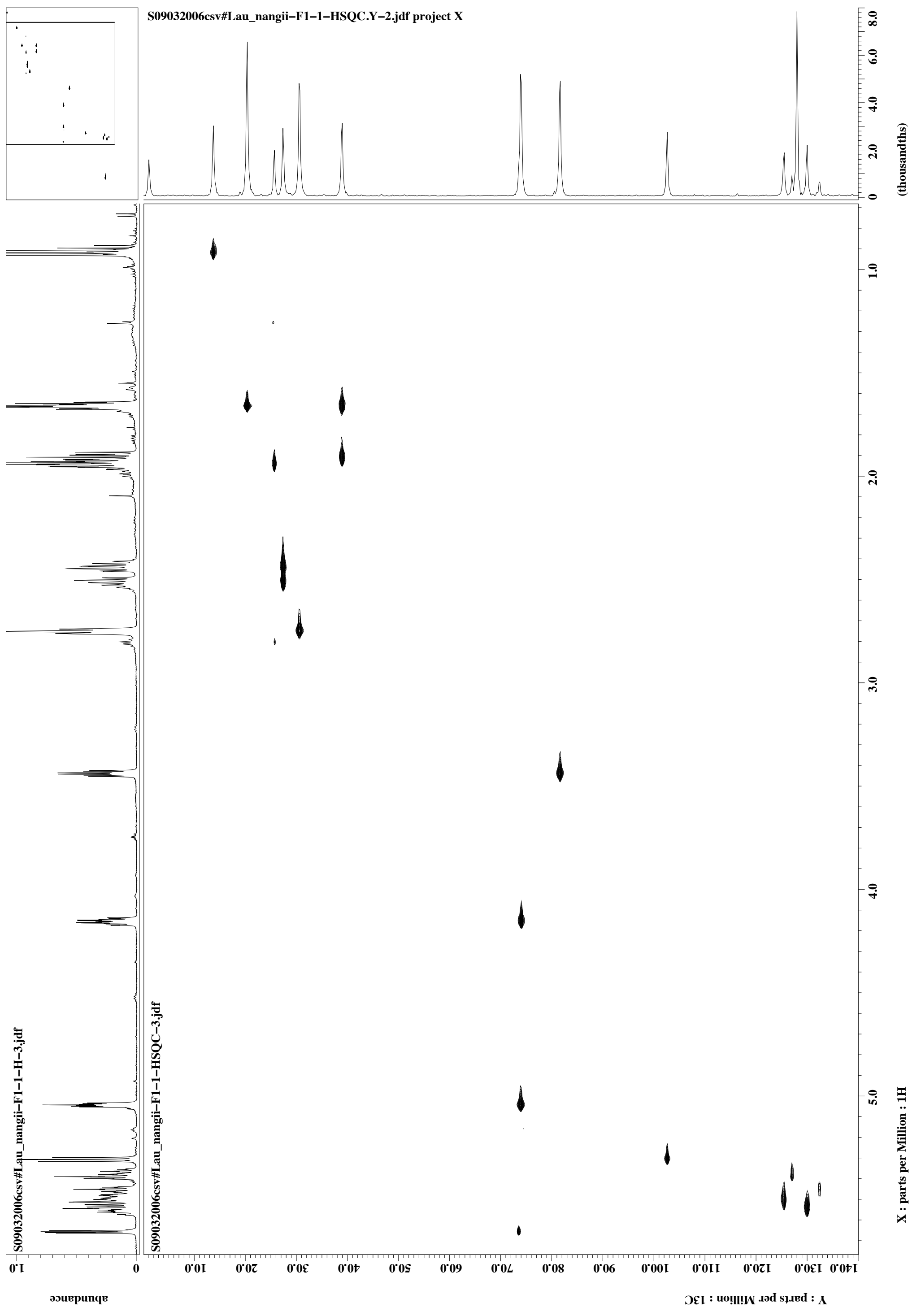

HSQC COMPOUND 2

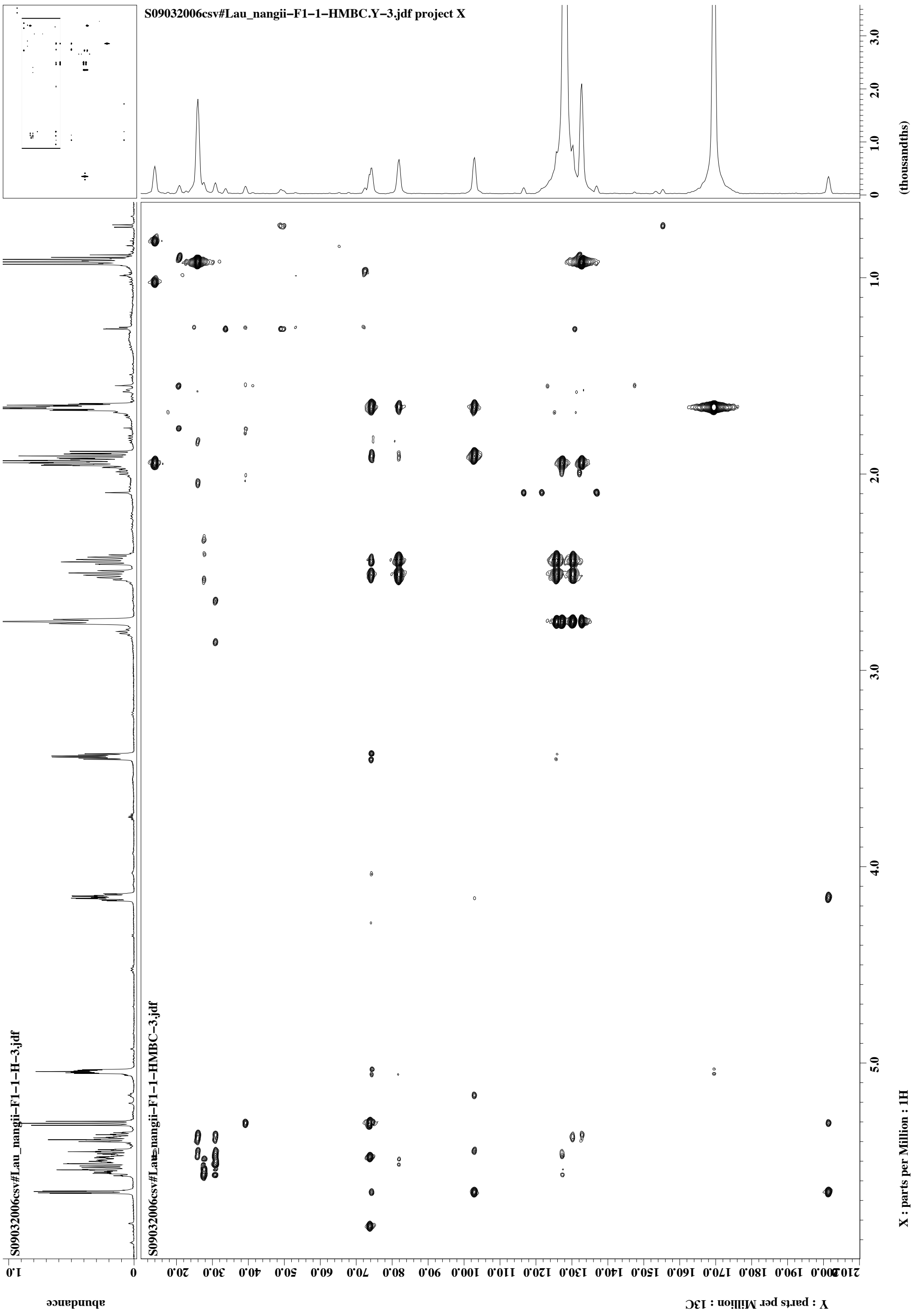

HMBC COMPOUND 2
